# Supplementary figures and images for: Photocatalysis and adsorption kinetics of azo dyes by nanoparticles of nickel oxide and copper oxide and their nanocomposite in an aqueous medium
Source: PeerJ. 2022 Nov 14;10:e14358. doi: 10.7717/peerj.14358 (PMC9671035; doi:10.7717/peerj.14358)

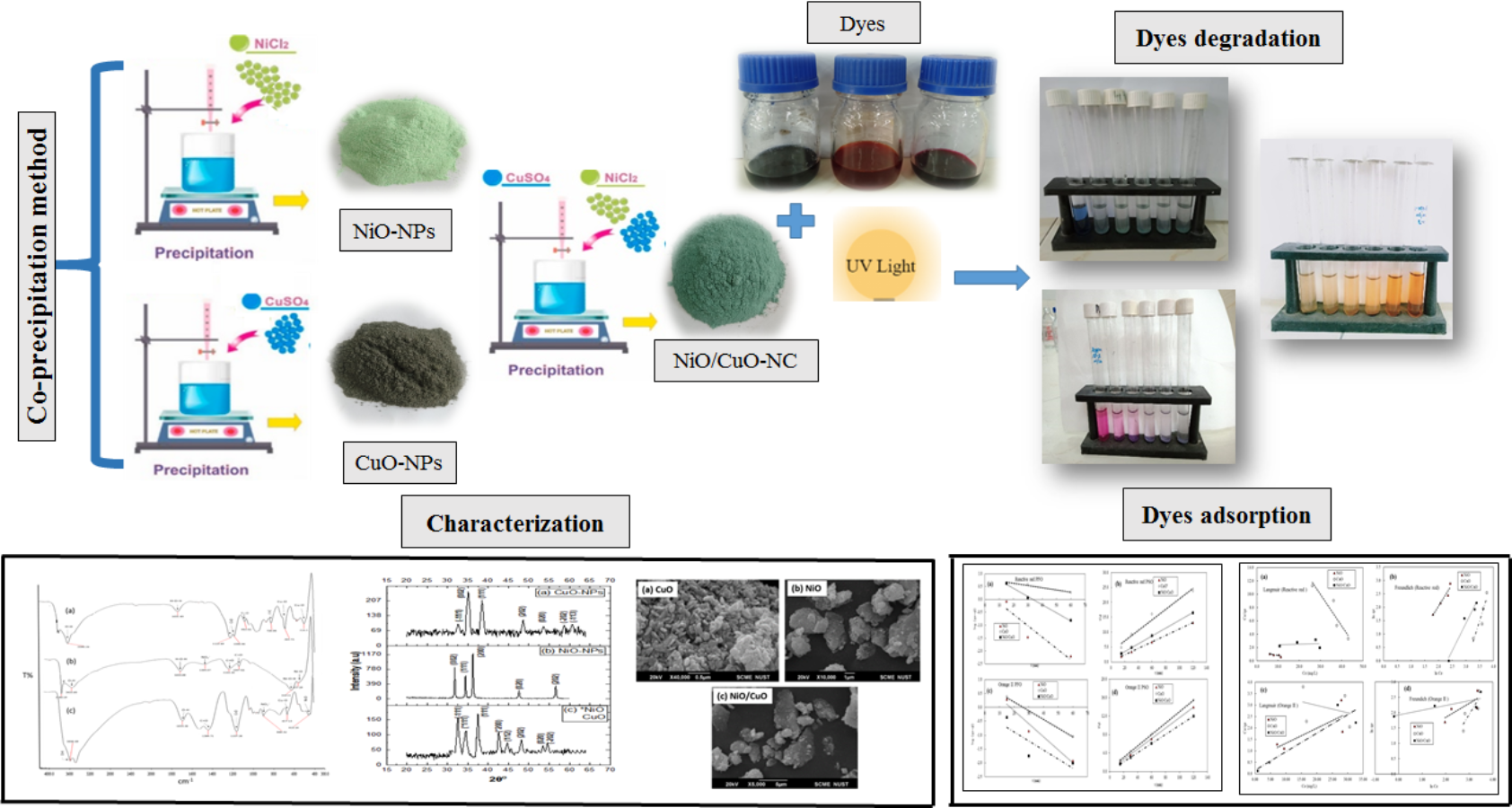

Supplement: Supplemental Information 2 [file peerj-10-14358-s002.png]
